# Supplementary material for: The role of physical activity in modulating six-minute walk distance in adolescents
Source: PLoS One. 2026 Mar 17;21(3):e0344115. doi: 10.1371/journal.pone.0344115 (PMC12994793; doi:10.1371/journal.pone.0344115)
Supplement: Attachment — Submitted filename: Response to Reviewers.pdf [file pone.0344115.s003.pdf]

REVIEWER 1

We would like to thank the reviewer for their constructive feedback.

Introduction

- **Lines 47 & 56** - The dichotomy "healthy and clinical populations" is unclear. The term *clinical* is inappropriate here. Please specify which clinical conditions are being contrasted with healthy cohorts, or replace with more precise language.
  Authors reply: Thank you for your comment. The sentence has been slightly rephrased to use the term "healthcare settings", in line with the cited article.
- **Line 61** - The phrase "In the last years" is vague. Please specify the time period (e.g. "Between 2015 and 2022, the …").
  Authors reply: Thank you for your comment. The sentence has been rephrased to more clearly delineate the temporal scope of the test's application among specific populations.
- **Lines 54–69** - The categorical grouping of conditions (metabolic, pulmonary, cardiovascular, musculoskeletal) is incorrect (e.g., asthma is pulmonary, not metabolic or musculoskeletal). Revise or clarify to ensure each condition is referred to or classified correctly.
  Authors reply: Thank you for this useful comment. The part of the sentence referring to these conditions has been revised to enhance readability and improve the overall clarity related to these conditions.
- **Lines 72–74** - Change "Most considered variables" to "Variables with the greatest predictive weight" (or similar).
  Authors reply: Thank you for your comment. This part is now changed, and it reads "variables with the highest predictive significance".
- **Lines 88–89** - Revise "different contexts" to "different geographic or demographic contexts," since variation may occur within a single country.
  Authors reply: Thank you for the comment. This sentence is now revised, and it reads as follows: "Consequently, researchers strongly recommend establishing specific reference values for various population groups based on different geographic or demographic contexts".
- **Lines 88–96** - The terms "reference values," "equations," and "population standards" are used without sufficient contextualisation. Be more specific when referring to these factors – (e.g equations for what? population standards for what?). Explain what outcomes the equations predict, and cite recent literature justifying the need for improved 6MWD prediction models.
  Authors reply: Thank you for this useful comment. The sentence has been rephrased, and it clearly states what each of these terms refers to. The sentence now reads as follows: "In their review, Mylius et al. [16] compared data from multiple countries and found that reference values and predictive equations for the 6MWD in children and adolescents developed in one country may not be applicable or significant in others".
- **Lines 95–98** - The assertion that "better results were reported in middle-income countries" lacks substantiation. Provide references, acknowledge the absence of socioeconomic data in prior studies, and offer plausible explanations for observed differences.
  Authors reply: Thank you for your comments. The sentence has now been clarified by incorporating the respective values recorded in each country. Additionally, the potential reasons underlying the observed differences between the two countries have been addressed.
- **Lines 99–117** - The rationale for conducting this study in Italy is underdeveloped in this section. Please review relevant European or Italian adolescent health literature to establish the study's necessity and contextualize the findings. Furthermore, while the authors acknowledge the link between cardiovascular health and 6MWD – there is little rationale explaining why these two are relevant to the population understudy.
  Authors reply: Thank you for your comment. By measuring the 6MWD, this submaximal exercise test assesses aerobic capacity and endurance without pushing individuals to their absolute limit, thus making it ideal for adolescents. Although it is not a direct measure, 6MWD has shown a strong correlation with maximal oxygen consumption, which is considered a gold standard for cardiorespiratory fitness and, indeed, a relevant measure of cardiovascular health. Furthermore, in

this paragraph, we also intended to reflect on research done on a sample of younger participants (PMCID: PMC6188863). Given the absence of reported data on Italian adolescents in the existing literature, a significant gap remains in research addressing this population. Building on this foundation, we infer that there is a compelling rationale for extending the investigation of these parameters to older populations. An explanation of the importance of providing reference values was also added.

<u>Methods</u>

Measures

- **Line 139** - Define "CRF" (cardiorespiratory fitness) at first mention.
  Authors reply: Thank you for your suggestion; however, the CRF is defined in the introduction part. Hence, we agreed that there is no need to define it also in the Measures.

Statistical Analyses

- **Line 166** - Excluding participants without complete personal data may introduce selection bias. Acknowledge this in the limitations.
  Authors reply: Thank you for your comment. Full personal data referred to participants' age, weight, and height. Without these data, it was not possible to perform the main analyses. We apologise for not being completely clear about that. The sentence has been revised.
- **Line 169** - A non-normal distribution was noted, yet results are summarized using means and standard deviations. Report medians and interquartile ranges instead, or justify why mean ± SD are appropriate.
  Authors reply: Thank you for your comment. The results were presented as mean ± standard deviation since the mean and median showed similar values. The difference between median and mean was 0.19, -1.23, -0.09, -0.32, and -1.56 for age, weight, height, BMI and 6MWD, respectively. So, the mean values were not affected by outliers. Data are now presented according to the reviewer's suggestion.
- **Lines 169–180** - The use of a linear regression analysis appears unsubstantiated and requires elaboration. Given the non-normal distribution. the data is likely to violate several assumptions that are essential to consider when conducting a linear regression. Specify which regression assumptions were tested (e.g., normality of residuals, homoscedasticity), present these diagnostics in a supplement, and cite best practices for outlier removal and model selection criteria (AIC/BIC). It remains unclear whether all predictors were entered into a single regression model. Including highly correlated variables (height, weight, and BMI) violates the independence assumption, introduces multicollinearity, inflates standard errors, and undermines interpretability. If BMI is included alongside height and weight, the model essentially attempts to partition shared variance among highly correlated predictors, violating the assumption of independence among predictors and potentially leading to misleading conclusions. I strongly recommend revisiting the model either (a) modelling BMI alone or (b) modelling height and weight separately with age to determine interactions
  Authors reply: Thank you for your valuable feedback. It was a mistake. The regression assumptions were checked as follows. The normality of residuals was also checked by plotting the standardized residuals and the Standardized Predicted Values. As shown in the following figures, a good normality of residuals was found.

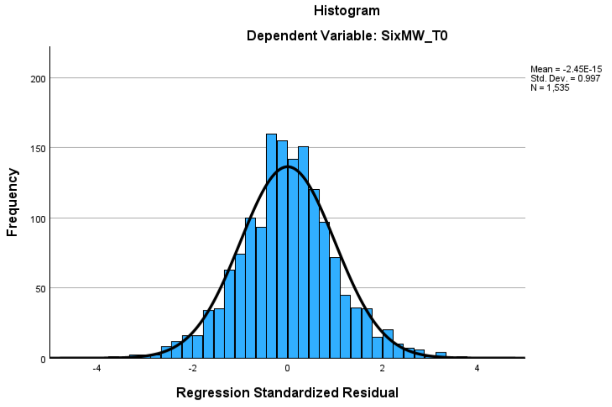

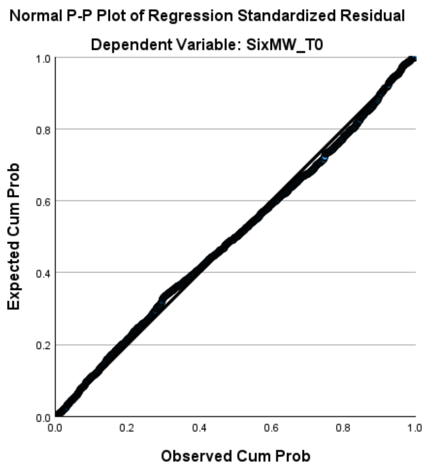

The multicollinearity was checked by calculating the Variance Inflation Factor (VIF) score, which was 1.7, 1.7, 1.1 and 1.0 for age, height, BMI and MVPA, respectively. Moreover, to better investigate possible multicollinearity between two highly related variables (BMI and height), Spearman's correlation was performed, showing a low positive Spearman's correlation (.344). Based on these results, the multicollinearity assumption was satisfied. The homoscedasticity was checked by performing the Breusch–Pagan/Cook–Weisberg test for heteroskedasticity. The results showed a violation of this regression assumption (p=.0019). For this reason, a Robust Standard Errors method was used.

In female group, the normality of residuals was also checked by plotting the standardized residuals and the Standardized Predicted Values. As shown in the following figures, a good normality of residuals was found.

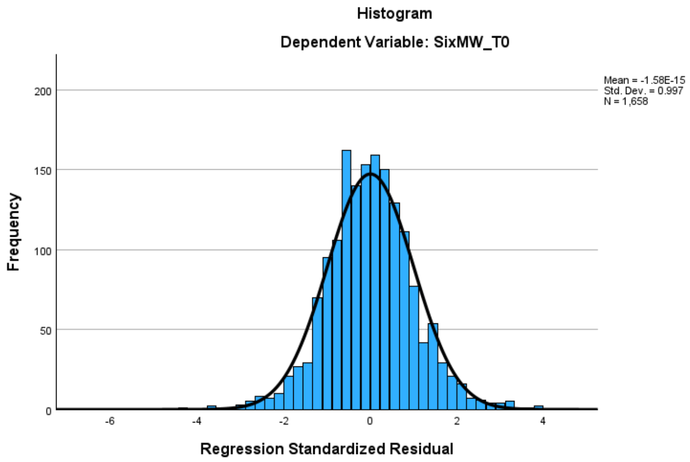

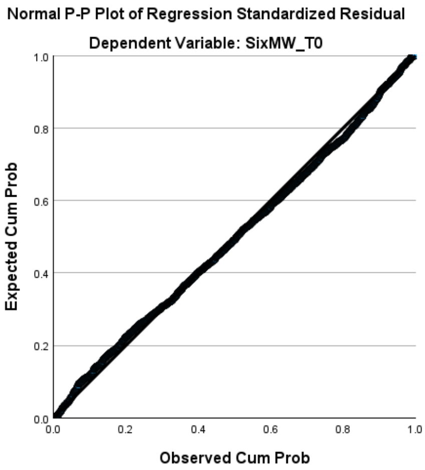

The multicollinearity was checked by calculating the Variance Inflation Factor (VIF) score, which was 1.5, 1.4, 1.1 and 1.0 for age, height, BMI and MVPA, respectively. Moreover, to better investigate possible multicollinearity between two highly related variables (BMI and height), Spearman's correlation was performed, showing a low positive correlation (.204). Based on these results, the multicollinearity assumption was satisfied.

The models, results and discussion have been modified according to reviewers' suggestions.

Results

- **Line 188 -** Clarify whether the reported BMI ("20.00 ± 3.20 kg/m²") is mean ± SD or median (IQR).
  Authors reply: The data were reported as mean ± SD. It is now expressed as median (IQR).
  Could the authors include more information on BMI and how it reflects overall health status in this context? As it stands, it is difficult to assess whether the individuals in the study can be considered healthy, especially given the lack of clinical or physiological markers beyond BMI.

Clarifying this would help readers better understand the health profile of the study population – and provide context to the utility of MVPA as a marker of CRF.
Authors reply: Thank you for your comment. We agree with the reviewer, it is essential to collect data relating to the physical, mental, emotional and social spheres to define a child, adolescent, adult or elderly person as healthy. Unfortunately, we did not collect this data. BMI could only be used to determine if a person is underweight, healthy weight, overweight, or obese. Moreover, as reported by the National Center for Health Statistics in collaboration with the National Center for Chronic Diseases Prevention and Health Promotion, the BMI cut-off values differ according to age and sex. Consequently, we can only describe the BMI status considering these two variables. Some additional information on the participants' BMI condition is now added in the results paragraph.

- **Line 190** - The statement regarding WHO-recommended MVPA levels belongs in the Discussion.
  Authors reply: Thank you, we appreciate this comment. The part with the WHO recommendations for MVPA is now included in the second paragraph of the discussion. It has been slightly revised to more clearly highlight the significance of these percentages, as well as the walking distances recorded for male and female participants.
- **Line 190** - Justify the exclusion of outliers by age group and provide details in a supplement.
  Authors reply: As described in the statistical analysis section, boxplots method was used to detect outlier data of 6MWD and values > 3 IQR's from the end of the box were identified as outliers and were excluded from the data analyses. The 1.5 IQR rule is a widely accepted as conventional method for outlier detection. By extending this to a 3 IQR rule, a more conservative approach was adopted (doi: 10.1080/10691898.2011.11889610). This method was chosen because it's a non-parametric approach that is less sensitive to extreme values compared to methods based on the mean and standard deviation. By applying this criterion, only the most extreme data points, which could potentially skew our results, were excluded from the final analysis, avoiding the accidental exclusion of valid data points that are merely on the tail ends of the distribution. The sentence has been modified to improve clarity.
- **Line 201** - When reporting sex differences in 6MWD, specify whether these differences apply across all ages or vary by age subgroup.
  Authors reply: Thank you for your comment. Speaking generally, differences between males and females in youth are very common, especially in terms of cardiorespiratory fitness (doi:10.1161/CIR.0000000000000866, doi: 10.5604/01.3001.0053.7363, doi: 10.3389/fped.2021.657551). Potential explanations for this difference include sex-related differences in muscle fibre type, oxygen extraction, or the lipid content of myofibrils (doi: 10.1016/j.jsams.2008.05.006, doi: 10.1152/ajpregu.00472.2006). To answer the reviewer's comment, we performed the analyses to investigate whether the differences between the two sexes apply across all ages. After the assumptions check, linear regression with robust standard errors was performed. Results showed a significant interaction between gender and age (B: -11.027, t: -7.45, p<0.001, CI: -13.929 – -8.126). However, since these analyses extend beyond the primary scope of the study, we have jointly decided to omit them from the manuscript.
- **Line 214** - Clarify whether "6MWT" differs from "6MWD" or if the terms are used interchangeably.
  Authors reply: Thank you for your comment. Although 6MWT and 6MWD are closely related, they refer to different aspects of the same assessment. 6MWT – stands for the standardized submaximal test protocol used to assess the functional capacity. 6MWD is a result of this test – total distance walked during the 6 minutes usually expressed in meters (m). To put it simple way: 6MWT is the test and 6MWD is the score.
- **Figure 1a & 1b** - Add individual data points or provide supplementary scatterplots to allow readers to assess distribution and potential clustering.
  Authors reply: Thank you for your suggestion. The graphs have been changed.

Discussion

- **Lines 223–240** – The authors need to re-evaluate their models, and ensure their discussion addresses possible interaction effects.

Authors reply: Thank you for your comment. Based on the previous suggestions, we have re-evaluated the models and the possible interaction effects are now addressed.

- **Lines 250–251** - When comparing your results to those from Acuri et al. (2016) and other countries, discuss potential cultural, socioeconomic, or environmental factors that may account for observed similarities or differences between Italian adolescents and peers in Switzerland, Tunisia, Austria, and Brazil.
  Authors reply: Thank you for your comment. We agree that these similarities and differences could have been influenced by cultural, socioeconomic and environmental factors. However, neither Arcuri et al. (2016) nor other authors reporting results from their respective countries have thoroughly examined these factors in their work. While these variables may hold relevance, they were not investigated as part of the present study. Therefore, we could not discuss these factors in detail.
- **Line 273** - The phrase "state of physical fitness" is vague. Specify whether you refer to objectively measured fitness, self-reported activity levels, or other proxies, and discuss potential biases or population differences.
  Authors reply: Thank you for your comment. This is a very good point. In this context, physical fitness refers to each participant's individual fitness level. It represents a personal state shaped by daily lifestyle habits, rather than a directly measured variable. For instance, while some individuals may engage in regular leisure-time physical activity, others may not, reflecting natural variation in everyday behaviour.
- **Line 274 & 283–285** - Define "PA" (physical activity) when first used. Consider interactions between MVPA and BMI in your models - does BMI moderate the effect of MVPA on 6MWD?
  Authors reply: Thank you very much for your comment. Physical activity (PA) is now defined at its first mention in the text, ensuring clarity for the reader throughout the document. Performing the suggested models, results showed no interaction effects.
  Also discuss how gendered social norms, environmental opportunities, and motivational factors might influence activity levels.
  Authors reply: Thank you very much for your comment. We really appreciate the idea of discussing the gendered social norms, environmental opportunities, motivational factors and their possible effect on activity levels. However, as the aim of the study was to establish the reference values of 6MWD in adolescents, we argue that broadening the work in this direction could divert the attention of the reader from the central objective. We also strongly agree that these factors represent complex parameters that warrant independent investigation. Each could serve as the foundation for a separate study, particularly one focused on examining their influence on PA levels.
- **Interpretation of Correlations** - The positive correlations of age, height, and BMI with 6MWD in males (versus negative in females) warrant deeper biological, social, and cultural contextualization in light of adolescent growth trajectories and gender norms relating to health and fitness.
  Authors reply: Thank you for your comment. This is an important point for discussion. The section addressing gender discrepancies has now been expanded to offer a plausible explanation for the observed results, given that these influential factors were not directly measured in the present study.
- **MPVA Findings** - Discuss why MVPA levels fell below WHO recommendations despite the study population being a seemingly healthy cohort.
  Authors reply: Thank you for your comment. This is a highly relevant issue to address, particularly given that adolescence is a sensitive and formative period. During this stage of life, adolescents may not fully grasp the long-term health benefits of regular MVPA. As a result, the absence of immediate, visible outcomes can lead to a lack of motivation to engage in physical activity during leisure time. Additionally, urban environments often lack accessible and safe spaces for outdoor activities, further limiting opportunities for movement. The widespread use of smartphones, streaming platforms, and video games has also contributed to an increasingly sedentary lifestyle among adolescents. Moreover, shifting priorities toward academic responsibilities, social interactions, and digital engagement may lead to physical activity being

deprioritized in daily routines of adolescents. While these variables may hold relevance, they were not investigated as part of the present study. Therefore, we could not discuss these factors in detail.

- **Adolescent 6MWD Decline** - Explore literature addressing the decline in 6MWD between ages 13–16, linking to pubertal development, motivational factors, or methodological considerations.
  Authors reply: Thank you for your comment. Although it is challenging to identify a specific cause for the observed decline in 6MWD between ages 13-16, we have discussed several possible contributing factors in the discussion paragraph.

Limitations

- Several limitations are not addressed, including selection bias from data exclusions, potential measurement error in MVPA assessment, and the generalizability of findings beyond a high-income, European context.
  Authors reply: Thank you for your comment. The limitations section has been slightly expanded to address these points more thoroughly.
- Furthermore, while the authors mention some limitations they do not elaborate on how these may have influenced the study's design or findings.
  Authors reply: Thank you for your comment. The part related to the study limitations is now modified accordingly and better depicts the limitations of the present work.

REVIEWER 2

We would like to thank the reviewer for their constructive feedback.

Reviewer comments

- Authors covered a gap in the literature related to the normative data in Italian adolescents about 6MWT. The sample is big and representative of the agegroup. However it has been colleceted only in one region as mentioned as limitation by the authors. I have some doubt about the self report measure. Authgors should spend some more words in the discussion about the limitation of the study, since it has been reported in the cited paper (Booth; the physical activity questions in the WHO health behaviour in schoolchildren (HBSC) survey) that there is an agrement from 70% to 85% in test retest. Moreover some references about the reliability with objective measure should be reported. Also self reporting heigth and weigth could introduce biases.
  Authors reply: Thank you for your comment. The part related to the limitations of the study has been slightly modified, addressing more clearly the limitations of the present work.
- In table 2 it is not reported the regression for age in males. Please add.
  Authors reply: Thank you for your suggestion. The table was created to show only the statistically significant results.

Journal requirements

Journal requirements:
When submitting your revision, we need you to address these additional requirements.
1. Please ensure that your manuscript meets PLOS ONE's style requirements, including those for file naming. The PLOS ONE style templates can be found at https://journals.plos.org/plosone/s/file?id=wjVg/PLOSOne_formatting_sample_main_body.pdf and https://journals.plos.org/plosone/s/file?id=ba62/PLOSOne_formatting_sample_title_authors_affiliations.pdf
Authors reply: Done.

2. Thank you for stating the following financial disclosure:
"This study was funded by the Free University of Bozen-Bolzano, grant No. 6118, as a part of the research project "PE4MOVE - L'educazione Fisica per la promozione dell'attività motoria e sportiva degli studenti nel tempo extrascolastico" [Physical Education to Promote Out-of-School Physical Activity]. The paper publication was also supported by the Open Access Publishing Fund of the Free University of Bozen-Bolzano."
Please state what role the funders took in the study. If the funders had no role, please state: "The funders had no role in study design, data collection and analysis, decision to publish, or preparation of the manuscript."
Authors reply: the statement "The funders had no role in study design, data collection and analysis, decision to publish, or preparation of the manuscript.", was added.

If this statement is not correct you must amend it as needed.
Please include this amended Role of Funder statement in your cover letter; we will change the online submission form on your behalf.
Authors reply: Done.

3. We note that you have indicated that there are restrictions to data sharing for this study. For studies involving human research participant data or other sensitive data, we encourage authors to share de-identified or anonymized data. However, when data cannot be publicly shared for ethical reasons, we allow authors to make their data sets available upon request. For information on unacceptable data access restrictions, please see http://journals.plos.org/plosone/s/data-availability#loc-unacceptable-data-access-restrictions.
Before we proceed with your manuscript, please address the following prompts:
a) If there are ethical or legal restrictions on sharing a de-identified data set, please explain them in detail (e.g., data contain potentially identifying or sensitive patient information, data are owned by a third-party organization, etc.) and who has imposed them (e.g., a Research Ethics Committee or Institutional Review Board, etc.). Please also provide contact information for a data access committee, ethics committee, or other institutional body to which data requests may be sent.
b) If there are no restrictions, please upload the minimal anonymized data set necessary to replicate your study findings to a stable, public repository and provide us with the relevant URLs, DOIs, or accession numbers. Please see http://www.bmj.com/content/340/bmj.c181.long for guidelines on how to de-identify and prepare clinical data for publication. For a list of recommended repositories, please see https://journals.plos.org/plosone/s/recommended-repositories. You also have the option of

uploading the data as Supporting Information files, but we would recommend depositing data directly to a data repository if possible.
Authors reply: Done.

Please update your Data Availability statement in the submission form accordingly.
4. In the online submission form you indicate that your data is not available for proprietary reasons and have provided a contact point for accessing this data. Please note that your current contact point is a co-author on this manuscript. According to our Data Policy, the contact point must not be an author on the manuscript and must be an institutional contact, ideally not an individual. Please revise your data statement to a non-author institutional point of contact, such as a data access or ethics committee, and send this to us via return email. Please also include contact information for the third party organization, and please include the full citation of where the data can be found.
Authors reply: Done.

5. We note that there is identifying data in Tables 1a and 1b. Due to the inclusion of these potentially identifying data, we have removed this file from your file inventory. Prior to sharing human research participant data, authors should consult with an ethics committee to ensure data are shared in accordance with participant consent and all applicable local laws. Data sharing should never compromise participant privacy. It is therefore not appropriate to publicly share personally identifiable data on human research participants. The following are examples of data that should not be shared:
-Name, initials, physical address
-Ages more specific than whole numbers
-Internet protocol (IP) address
-Specific dates (birth dates, death dates, examination dates, etc.)
-Contact information such as phone number or email address
-Location data
-ID numbers that seem specific (long numbers, include initials, titled "Hospital ID") rather than random (small numbers in numerical order)
Data that are not directly identifying may also be inappropriate to share, as in combination they can become identifying. For example, data collected from a small group of participants, vulnerable populations, or private groups should not be shared if they involve indirect identifiers (such as sex, ethnicity, location, etc.) that may risk the identification of study participants.
Additional guidance on preparing raw data for publication can be found in our Data Policy (https://journals.plos.org/plosone/s/data-availability#loc-human-research-participant-data-and-other-sensitive-data) and in the following article: http://www.bmj.com/content/340/bmj.c181.long.
Please remove or anonymize all personal information, ensure that the data shared are in accordance with participant consent, and re-upload a fully anonymized data set. Please note that spreadsheet columns with personal information must be removed and not hidden as all hidden columns will appear in the published file.
Authors' reply: Table 1a and 1b did not show identifying data.

If the reviewer comments include a recommendation to cite specific previously published works, please review and evaluate these publications to determine whether they are relevant and should be cited. There is no requirement to cite these works unless the editor has indicated otherwise.
Authors reply: Thank you for your comment. It was considered during the review process.
