# Supplementary material for: The role of physical activity in modulating six-minute walk distance in adolescents
Source: PLoS One. 2026 Mar 17;21(3):e0344115. doi: 10.1371/journal.pone.0344115 (PMC12994793; doi:10.1371/journal.pone.0344115)
Supplement: Attachment — Submitted filename: Response_to_Reviewers_auresp_2.pdf [file pone.0344115.s004.pdf]

REVIEWER 1

I would like to commend the authors of this manuscript, and on their efforts to answer and address all the questions from my review. There are three small comments that I would like to see addressed, and then I would be more than happy to recommend this for publication:

Authors' reply: We sincerely thank Reviewer 1 for the kind words and for taking the time to provide thoughtful feedback on our manuscript. We have carefully considered your three additional comments and have revised the manuscript accordingly.

1. Line 186: The authors reference a "robust standard errors method". Please include a reference for this.

Authors' reply: We thank the reviewer for this comment. We have now included a reference for the robust standard errors method (White, 1980), and the Methods section has been updated accordingly. We hereby note that the reference has been added to the manuscript as reference [32]; doi.org/10.2307/1912934.

2. Line 199: Revise the wording 'highest percentage of people (26.7%)' - this amount cannot be the highest percentage of people, and should be worded differently.

Authors' reply: We thank the reviewer for this suggestion. The wording has been revised accordingly, and the manuscript now states as follows: "The most frequently reported level of physical activity was 2–3 hours per week (26.7%)."

3. Inclusion of some of the research cited in the authors responses:

3a. In response to my comment regarding specifying whether sex differences occur by age the authors provide rich context as to the possible biological sex-related differences and this would enrich the introduction/discussion if included. Specifically, "Speaking generally, differences between males and females in youth are very common, especially in terms of cardiorespiratory fitness (doi:10.1161/CIR.0000000000000866, doi: 10.5604/01.3001.0053.7363, doi: 0.3389/fped.2021.657551). Potential explanations for this difference include sex-related differences in muscle fibre type, oxygen extraction, or the lipid content of myofibrils (doi: 10.1016/j.jsams.2008.05.006, doi: 10.1152/ajpregu.00472.2006)."

Authors' reply: We thank the reviewer for this valuable suggestion. The context regarding possible biological sex-related differences has now been incorporated into the Discussion section, as recommended.

We hereby note that the following references have been added to the manuscript:

[4]; doi.org/10.1161/CIR.0000000000000866

[38]; doi:10.5604/01.3001.0053.7363

[39]; doi:10.14814/phy2.70616

[40]; doi:10.1016/j.jsams.2008.05.006

[41]; doi:10.1152/ajpregu.00472.2006

3b. In response to my comment :'Discuss why MVPA levels fell below WHO recommendations despite the study population being a seemingly healthy cohort".

The authors state: "As a result, the absence of immediate, visible outcomes can lead to a lack of motivation to engage in physical activity during leisure time. Additionally, urban environments often lack accessible and safe spaces for outdoor activities, further limiting opportunities for movement. The widespread use of smartphones, streaming platforms, and video games has also contributed to an increasingly sedentary lifestyle among adolescents. Moreover, shifting priorities toward academic responsibilities, social interactions, and digital engagement may lead to physical activity being deprioritized in daily routines of adolescents. While these variables may hold relevance, they were not investigated as part of the present. While these variables may hold relevance, they were not investigated as part of the present study. Therefore, we could not discuss these factors in detail". I believe the findings in your article would be enriched by including the context provided in your response to my comment.

Authors' reply: We thank the reviewer for this constructive suggestion. The contextual discussion regarding potential motivational, environmental, and lifestyle factors influencing physical activity has now been incorporated into the Discussion section of the manuscript. These factors are presented as plausible explanations and are clearly framed as not having been directly assessed in the present study.

We hereby note that another reference has been added to the manuscript as reference [42]; doi:10.3389/fphys.2023.1131195.

REVIEWER 2

Authors have adressed my comments. I do not have any other comment and suggest acceptance of the manuscript.

Authors' reply: We sincerely thank Reviewer 2 for the careful evaluation of our manuscript and for the positive feedback. We greatly appreciate the time and effort devoted to reviewing our work and are grateful for the recommendation for acceptance.
