# Supplementary material for: The role of physical activity in modulating six-minute walk distance in adolescents
Source: PLoS One. 2026 Mar 17;21(3):e0344115. doi: 10.1371/journal.pone.0344115 (PMC12994793; doi:10.1371/journal.pone.0344115)
Supplement: Attachment — Submitted filename: Review_6MWD in Italian adolescents_2025.docx [file pone.0344115.s001.docx]

**Elizabeth Dinkele, PhD**

**University of Cape Town,**

**Anzio Road, Observatory, Cape Town, South Africa**

[**elizabethdinkele@gmail.com**](mailto:elizabethdinkele@gmail.com)

21 July 2025

Dear PLOS ONE editors,

Thank you for the opportunity to review the manuscript PONE-D-25-18111, “The role of physical activity in modulating six-minute walk distance in adolescents”. The article presents original research on the determinants of six-minute walk distance (6MWD) in adolescents. Several issues in clarity, terminology, methodological reporting, and contextualization must be addressed before the manuscript is suitable for publication. My specific comments are organized by manuscript section and line number below.

**Introduction**

1. **Lines 47 & 56** - The dichotomy “healthy and clinical populations” is unclear. The term *clinical* is inappropriate here. Please specify which clinical conditions are being contrasted with healthy cohorts, or replace with more precise language.
2. **Line 61** - The phrase “In the last years” is vague. Please specify the time period (e.g. “Between 2015 and 2022, the …”).
3. **Lines 54–69** - The categorical grouping of conditions (metabolic, pulmonary, cardiovascular, musculoskeletal) is incorrect (e.g., asthma is pulmonary, not metabolic or musculoskeletal). Revise or clarify to ensure each condition is referred to or classified correctly.
4. **Lines 72–74** - Change “Most considered variables” to “Variables with the greatest predictive weight” (or similar).
5. **Lines 88–89** - Revise “different contexts” to “different geographic or demographic contexts,” since variation may occur within a single country.
6. **Lines 88–96** - The terms “reference values,” “equations,” and “population standards” are used without sufficient contextualisation. Be more specific when referring to these factors – (e.g equations for what? population standards for what?). Explain what outcomes the equations predict, and cite recent literature justifying the need for improved 6MWD prediction models.
7. **Lines 95–98** - The assertion that “better results were reported in middle-income countries” lacks substantiation. Provide references, acknowledge the absence of socioeconomic data in prior studies, and offer plausible explanations for observed differences.
8. **Lines 99–117** - The rationale for conducting this study in Italy is underdeveloped in this section. Please review relevant European or Italian adolescent health literature to establish the study’s necessity and contextualize the findings. Furthermore, while the authors acknowledge the link between cardiovascular health and 6MWD – there is little rationale explaining why these two are relevant to the population understudy.

**Methods**

**Measures**

- **Line 139** - Define “CRF” (cardiorespiratory fitness) at first mention.

**Statistical Analyses**

1. **Line 166** - Excluding participants without complete personal data may introduce selection bias. Acknowledge this in the limitations.
2. **Line 169** - A non-normal distribution was noted, yet results are summarized using means and standard deviations. Report medians and interquartile ranges instead, or justify why mean ± SD are appropriate.
3. **Lines 169–180** - The use of a linear regression analysis appears unsubstantiated and requires elaboration. Given the non-normal distribution. the data is likely to violate several assumptions that are essential to consider when conducting a linear regression. Specify which regression assumptions were tested (e.g., normality of residuals, homoscedasticity), present these diagnostics in a supplement, and cite best practices for outlier removal and model selection criteria (AIC/BIC).

It remains unclear whether all predictors were entered into a single regression model. Including highly correlated variables (height, weight, and BMI) violates the independence assumption, introduces multicollinearity, inflates standard errors, and undermines interpretability. If BMI is included alongside height and weight, the model essentially attempts to partition shared variance among highly correlated predictors, violating the assumption of independence among predictors and potentially leading to misleading conclusions. I strongly recommend revisiting the model either (a) modelling BMI alone or (b) modelling height and weight separately with age to determine interactions.

**Results**

1. **Line 188** - Clarify whether the reported BMI (“20.00 ± 3.20 kg/m²”) is mean ± SD or median (IQR). Could the authors include more information on BMI and how it reflects overall health status in this context? As it stands, it is difficult to assess whether the individuals in the study can be considered healthy, especially given the lack of clinical or physiological markers beyond BMI. Clarifying this would help readers better understand the health profile of the study population – and provide context to the utility of MVPA as a marker of CRF.
2. **Line 190** - The statement regarding WHO-recommended MVPA levels belongs in the Discussion.
3. **Line 190** - Justify the exclusion of outliers by age group and provide details in a supplement.
4. **Line 201** - When reporting sex differences in 6MWD, specify whether these differences apply across all ages or vary by age subgroup.
5. **Line 214** - Clarify whether “6MWT” differs from “6MWD” or if the terms are used interchangeably.
6. **Figure 1a & 1b** - Add individual data points or provide supplementary scatterplots to allow readers to assess distribution and potential clustering.

**Discussion**

1. **Lines 223–240** – The authors need to re-evaluate their models, and ensure their discussion addresses possible interaction effects.
2. **Lines 250–251** - When comparing your results to those from Acuri et al. (2016) and other countries, discuss potential cultural, socioeconomic, or environmental factors that may account for observed similarities or differences between Italian adolescents and peers in Switzerland, Tunisia, Austria, and Brazil.
3. **Line 273** - The phrase “state of physical fitness” is vague. Specify whether you refer to objectively measured fitness, self-reported activity levels, or other proxies, and discuss potential biases or population differences.
4. **Line 274 & 283–285** - Define “PA” (physical activity) when first used. Consider interactions between MVPA and BMI in your models - does BMI moderate the effect of MVPA on 6MWD? Also discuss how gendered social norms, environmental opportunities, and motivational factors might influence activity levels.
5. **Interpretation of Correlations** - The positive correlations of age, height, and BMI with 6MWD in males (versus negative in females) warrant deeper biological, social, and cultural contextualization in light of adolescent growth trajectories and gender norms relating to health and fitness.
6. **MPVA Findings** - Discuss why MVPA levels fell below WHO recommendations despite the study population being a seemingly healthy cohort.
7. **Adolescent 6MWD Decline** - Explore literature addressing the decline in 6MWD between ages 13–16, linking to pubertal development, motivational factors, or methodological considerations.

**Limitations**

- Several limitations are not addressed, including selection bias from data exclusions, potential measurement error in MVPA assessment, and the generalizability of findings beyond a high-income, European context.
- Furthermore, while the authors mention some limitations they do not elaborate on how these may have influenced the study’s design or findings.

**Conclusion**

The manuscript addresses a valuable research question, but substantial revisions are needed to clarify terminology, strengthen methodological transparency, ensure statistical validity, and properly contextualize. I would be happy to review a revised version of the manuscript that addresses these concerns.

**Warm regards**

**Elizabeth Dinkele, PhD.**
